# Ubiquitous Geo-Sensing for Context-Aware Analysis: Exploring Relationships between Environmental and Human Dynamics

**DOI:** 10.3390/s120709800

**Published:** 2012-07-18

**Authors:** Günther Sagl, Thomas Blaschke, Euro Beinat, Bernd Resch

**Affiliations:** 1 Doctoral College Geographic Information Science, University of Salzburg, Schillerstrasse 30, 5020 Salzburg, Austria; 2 Centre for Geoinformatics, University of Salzburg, Schillerstrasse 30, 5020 Salzburg, Austria; E-Mails: thomas.blaschke@sbg.ac.at (T.B.); euro.beinat@sbg.ac.at (E.B.); 3 Institute for Geoinformatics and Remote Sensing, University of Osnabrück, Barbarastrasse 22b, 49076 Osnabrück, Germany; E-Mail: bernd.resch@uni-osnabrueck.de; 4 SENSEable City Lab, Massachusetts Institute of Technology, 9-209, 77 Massachusetts Avenue, Cambridge, MA 02139, USA

**Keywords:** ubiquitous sensing, collective sensing, environmental monitoring, context awareness, sensor data, human-environmental interaction, spatio-temporal dynamics, urban dynamics, maximal information coefficient, geographic information science

## Abstract

Ubiquitous geo-sensing enables context-aware analyses of physical and social phenomena, *i.e.*, analyzing one phenomenon in the context of another. Although such context-aware analysis can potentially enable a more holistic understanding of spatio-temporal processes, it is rarely documented in the scientific literature yet. In this paper we analyzed the collective human behavior in the context of the weather. We therefore explored the complex relationships between these two spatio-temporal phenomena to provide novel insights into the dynamics of urban systems. Aggregated mobile phone data, which served as a proxy for collective human behavior, was linked with the weather data from climate stations in the case study area, the city of Udine, Northern Italy. To identify and characterize potential patterns within the weather-human relationships, we developed a hybrid approach which integrates several spatio-temporal statistical analysis methods. Thereby we show that explanatory factor analysis, when applied to a number of meteorological variables, can be used to differentiate between normal and adverse weather conditions. Further, we measured the strength of the relationship between the ‘global’ adverse weather conditions and the spatially explicit effective variations in user-generated mobile network traffic for three distinct periods using the Maximal Information Coefficient (MIC). The analyses result in three spatially referenced maps of MICs which reveal interesting insights into collective human dynamics in the context of weather, but also initiate several new scientific challenges.

## 1. Introduction

Understanding the complex interface between the environment and humans and its inherent dynamics is a multidisciplinary challenge. One precondition to achieving such knowledge is in any case the availability of representative data samples regarding the phenomena of interest. Ubiquitous sensing, or ubiquitous ‘geo-’sensing to emphasize the spatial dimension, and its underlying technologies provide such representative data even in real-time. Since citizens play a central role in urban dynamics, extensive scientific research is dedicated to the understanding of both individual and collective human behavior on different operational scales. One frequently used data source is user-generated traffic in mobile communication networks, which can be used to better understand and model collective human activity and mobility [1–3]. Furthermore, peoples' living environments are monitored and assessed by a variety of geospatial technologies such as remote sensing and especially *in-situ* sensor networks [4–6].

However, ‘what is really needed is a better understanding of human-environmental processes, *i.e.*, direct measures of the impact of human activities on the environment and direct measures of environmental stressors on human functions’ ([4], p. 1762). In this paper we address the latter research need by evaluating the spatio-temporal relationships between collective human dynamics and weather in an urban environment. We extend our hybrid approach [7] and apply inductive and deductive reasoning methods in order to evaluate the spatio-temporal patterns of human activity in Udine (Italy), taking into account the local weather.

Various different external or context factors within the domain of urban dynamics have been addressed in scientific literature (see next section: related work). Everyday experiences show that certain weather conditions may cause changes, unusual variations or even ‘anomalies’ in the patterns of individual and also collective mobility or activity. For instance, the sudden onset of heavy rainfall may instantaneously impact people's use of private or public transportation, or affect their (re-) arrangement of personal and business activities *etc.* However, the integration of environmental factors in general and meteorological conditions in particular into human dynamics analyses is rarely discussed. Untreated research questions comprise, for instance, what trends would emerge if we shift our attention to larger time scales, *i.e.*, zooming out temporally at a constant spatial scale? For example, during a persistent cold front or in the winter season, would the human mobility and activity patterns remain the same or would they change? Further, we could examine to what extent they would change and why and whether or not further driving factors play a role. One may continue zooming out, in both time and space, and observe yet other associations of environmental phenomena with human behavior even at a global scale and in the long-term. For instance, researchers have explored the association of El Niño/Southern Oscillation (ENSO) with civil conflicts [8], or climate change with large-scale mobility and migration [9]. With this paper we tackle this fundamental lack of understanding the impact of weather and climate conditions to human behavior by investigating meteorological and human dynamics at the operational scale of a small European city.

From a ubiquitous geo-sensing point of view, the understanding of the complex relationships between collective human activity and weather patterns can potentially provide novel insights into the underlying processes or cause-effect relationships. Such understanding can create spatio-temporal and situational awareness for various purposes, e.g., for emergency managers in terms of time-critical decision support, for public transportation operators in terms of efficient allocation of resources to enhance the traffic flow, for sales managers to support business intelligence, or for city planners regarding sustainable urban development. We therefore took environmental factors into account, in this case weather conditions, when modeling the human dynamics.

The following research question thus arises: can the multidimensional analysis of a joint data base, which consolidates weather data with mobile phone usage, reveal novel insights into the collective human dynamics in the context of weather, and thus into the complex human-environment interface?

In this paper we demonstrate that the weather-aware sensing of collective human activity can disclose additional insights into the complex human-environment interface. Therefore we verified and extended our approach described in [7] and consolidated the meteorological sensor measurements from environmental monitoring stations with human activity patterns, which were derived from user-generated mobile network traffic, on a common space-time basis. In order to explore temporal, spatial and periodic relationships within the comprehensive data set, we applied analysis methods from the time-, space-, and frequency domain. We also demonstrated that explanatory factor analysis can be used to extract basic weather conditions from a number of meteorological variables. Considering a real-time sensor data stream, this can reduce computation demands and thus significantly speed up near real-time weather analysis. Finally we evaluated spatio-temporal patterns in the relationships between effective local variations in mobile phone usage and adverse weather conditions using the maximal information coefficient as a measure of the strength of linear or non-linear association.

This paper is structured as follows: Section 2 discusses relevant and related work. Section 3 explains the methodology in our hybrid approach. Section 4 describes the case study setting including the data sets used. Section 5 presents the results followed by a critical discussion in Section 6. Section 7 addresses some limitations and constraints. Finally, in Section 8, we provide some conclusions and future research activities.

## 2. Related Work

The use of mobile phone data as a proxy for human activity patterns has already proved successful in several investigations [1–3]. Nonetheless, it is not clear if the sheer amount of mobile phone activity within certain areas and between cells may be correlated to environmental parameters. If this hypothesis holds true, then such investigations may provide some form of prediction of collective human activity in greater detail—with past data as in this paper, or even real time data in the future.

### 2.1. Human Dynamics and Context

In spite of the increasing interest in scientific literature, clear limitations exist in predicting human mobility using mobile phone data. For instance, the smallest units of investigation are cells. They can be considered as tessellated spaces of the estimated signal coverage of radio antennas [10]. They are usually not differentiated further in terms of geographic features such as land use and other points of interest [11].

Research so far has focused on particular contexts when analyzing human dynamics. For instance, and in addition to the approaches mentioned above, a recent body of literature in mobile phone research can be dedicated to three major context clusters in regard to human actions: mobility (e.g., [12,13]), activity in urban spaces (e.g., [14,15]), and (cross-border) social interactivity (e.g., [16,17]). Such human action patterns of both individuals [18] and communities [19] have been evaluated in the context of urban planning [14,20], or transportation [21]. In the context of scale, the human-city interaction system developed by Martino *et al.* [22] provides an interactive visualization of human movements across multiple temporal and spatial scales. Calabrese *et al.* [23] consider the context of social events and demonstrate that residents are more attracted to events if these take place close to their home location, for the Boston metropolitan area (MA, USA). Phithakkitnukoon *et al.* [18] found out that specific daily patterns of human activity strongly correlate with certain geographic areas that share a common characteristic context (e.g., shopping, eating). Hence, we conclude that the awareness of the respective context is significant when analyzing citizens' mobility and activity patterns.

### 2.2. Urban Dynamics and Environmental Factors

Today, ubiquitous sensing technologies and pervasive communication networks foster the digital earth concept introduced in 1998 [24]. This concept includes the continuous sensing and monitoring of physical and social phenomena. Given the growing number, and increasing capabilities of environmental sensors and sensor networks, the accuracy of the assessment of environmental parameters such as air temperature, precipitation, concentration of trace gases such as CO_2_, fine dust *etc.* is increasing at any scale. This also allows more accurate weather forecasting. On the other hand, two significant sources for collective social sensing exist [25]: (1) user generated traffic in wireless communication networks like the mobile phone network; (2) vast volumes of volunteered geographic information (VGI) [26] provided by individuals in e.g., social media. A combination of these two sources results in a digital replicate of social interactions and peoples' activity and mobility in remarkable spatial and temporal detail. Such ‘large-scale social sensors’ in combination with well-established environmental sensors and sensor networks provide comprehensive and even real-time data streams of our living space beyond simple snapshots. Moreover, investigating this digital data ocean by applying novel cross-domain analysis methods and data mining techniques could enhance process-understanding, and further enable processes-oriented modeling at different spatial and temporal scales.

Current projects from MIT's SENSEable City Lab, e.g., ‘LIVE Singapore!’, foster the vision of the real-time city by providing ‘a feedback loop between people, their actions, and the city’ [27]. Although suchlike projects integrate multiple and highly diverse data streams, also from social networks (e.g., Twitter), the consideration of ‘exogenous factors’ such as environmental conditions is still very rare. Yet, the only scientific work found that directly addresses the effects of weather on mobile social interaction, is a case study performed in Lisbon, Portugal [28]. By using one year of individual mobile phone data, and weather data, the authors compared the probability mass functions of average talk time, number of social ties, and number of strong/weak social ties to several classes per selected weather parameter (air temperature, relative humidity, air pressure, and wind speed). They conclude, among other things, that people tend to call longer when the air temperatures is low and the air pressure is high, and that people tend to communicate with fewer social ties when the weather conditions are uncomfortable. However, we identified two major shortcomings in that approach: firstly, it focuses on the attribute domain only, *i.e.*, it does not take into account the data sets' inherent spatial and temporal component, thus neglecting the spatio-temporal dynamics of the underlying phenomena (social interaction, and weather); secondly, the four meteorological parameters considered in the analysis are treated individually, *i.e.*, they do not describe ‘weather conditions’ (which is a higher level phenomenon). In contrast to that rather general approach, we preserve the spatio-temporal dynamics of the collective human behavior and the weather (including rainfall), and measure of the strength of the relationship between these two by using a novel statistic.

Hayes and Stephenson [29] combine physical sensor data with ‘online sensing’ data from social media sources (e.g., blogs, Twitter *etc.*) to determine deficiencies from physical sensors and sensor networks. Studies, such as [30,31], refer to future developments, where distributed geo-sensor networks in combination with Geographic Information Systems (GIS) infrastructures [32] will be employed to automatically generate multidimensional information close to real-time. This can enhance, for instance, the situational awareness for emergency management [33]. Such technological as well as methodological developments enable comprehensive insights into environmental processes and urban dynamics.

Within this research mobile phones are considered to be spatially distributed *in-situ* components of a large-scale sensor, thus being the mobile network as such. Although sensors such as digital compasses or gyroscopes embedded in today's smart phones are capable of detecting the user's immediate environment [34], we consider the entire mobile device as the sensing component. All these components are capable of disclosing the communication behavior and whereabouts of their users in a nonintrusive way. The fully anonymized and aggregated (temporally at 15 minute intervals; spatially at the location of the servicing antenna) user-generated traffic within the mobile network thus reflects the spatio-temporal activity and movement patterns of hundreds of thousands of subscribers.

## 3. Methodology

We validated our previous approach [7] and enhanced it by analyzing potential relationships of weather and mobile phone usage in multiple domains, *i.e.*, in addition to the frequency domain also in the time, and space domains.

### 3.1. From ‘Sensor’ Data to Information

We used different ‘sensing’ technologies in order to sense both environmental and human dynamics. On the one hand, we requested meteorological measurements from environmental monitoring stations to obtain the current state of the environment. On the other hand, we used user-generated mobile network data as a proxy to quantify human activity and mobility. Environmental sensor networks consist of multiple *in-situ* sensors, which measure a diversity of environmental parameters relating weather (temperature, precipitation *etc.*), air quality (trace gases, particulate matter *etc.*), hydrological conditions (river gauges, surface runoff *etc.*) and so on. Sensor nodes can include highly mobile and intelligent sensor pods [35], as well as fixed sensor stations [36]. Using measurements from these accurately calibrated sensors allows us to sense, monitor, and analyze the current state of the environment even close to real-time.

User-generated traffic in mobile phone networks, what we consider ‘large-scale sensors’, reflects the spatio-temporal activity of their subscribers. Depending on a provider's market share and mobile penetration rate, these patterns, to some degree, reflect the dynamics of the larger population.

### 3.2. Analysis Methods

We applied analysis methods from the time, space, and frequency domains in order to reveal potential patterns and relationships between weather and telecom data. Previous analysis methods in mobile phone traffic research included, for example, Eigen-decomposition [14] or multilevel regression analysis [19]. In contrast, we applied the following analysis methods to the multidimensional data set in order to unveil temporal, spatial, and cyclic patterns.

- Exploratory Factor Analysis of Meteorological Variables
- Bivariate Spectral Analysis
- Spatio-Temporal Extraction of Local Effective Variations in Mobile Network Traffic Intensity
- Maximal Information-Based Nonparametric Exploration of Adverse Weather Conditions and Effective Mobile Network Traffic Variations

In combination, these analysis methods build up our hybrid approach. The methods are described in more detail in the following sub-sections.

#### 3.2.1. Exploratory Factor Analysis of Meteorological Variables

We used Exploratory Factor Analysis (EFA) to reduce dimensionality and redundancy in a number of meteorological variables for the weather data. The projection of data to a lower-dimensional space (reduction of dimensionality) leads to a loss of the original data's individual structural relationships. However, since all of the considered meteorological variables contribute to describing the higher-level phenomenon, which is the weather, this loss of structure is in fact an advantage in terms of minimizing data redundancy. The resulting factors should account for simple weather conditions such as normal or adverse—as defined similarly in the context of failure rates in power transmission networks [37].

#### 3.2.2. Bivariate Spectral Analysis

A spectral analysis was performed in order to unveil the significant periodical components in the time series of the remaining factors, *i.e.*, in basic weather conditions (output of EFA), and mobile telecom traffic intensity. We applied a bivariate spectral analysis to highlight cross-spectral correlations between basic weather conditions and mobile phone traffic intensity. In order to evaluate the significance of such correlations the squared spectral coherence γ^2^—the spectral equivalent of the R^2^ in regression analysis [38]—was computed.

#### 3.2.3. Spatio-Temporal Extraction of Local Effective Variations in Mobile Network Traffic Intensity

**Figure 1.**
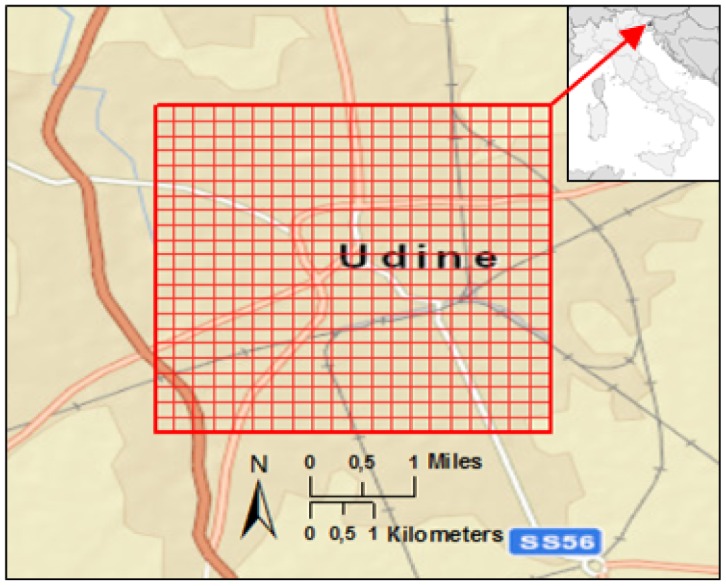
Study area: the urban environment of the city of Udine, Friuli Venetia Giulia Region, Italy; the red grid indicates the spatial resolution as 250 m × 250 m ‘pixels’ of the mobile network traffic.

We performed a high-resolution analysis to discover the effective variation from the expected value, which is the mean, in mobile network traffic intensity (this intensity is measured in Erlang, which is a dimensionless basic unit of telecom traffic intensity; it is named after A. K. Erlang: 1 Erlang equals 1 person calling 1 h, or 2 persons calling 0.5 h each, or three persons calling 20 minutes each and so on). In the ‘effective’ variations we excluded the day-night variations resulting from the general predominant day-night mobile network traffic intensity pattern. High-resolution refers to the spatial resolution of 250 m × 250 m (one ‘pixel’, as illustrated in Figure 1) and to the temporal resolution of 1 h of network traffic data being analyzed.

#### 3.2.4. Maximal Information-Based Nonparametric Exploration of Adverse Weather Conditions and Effective Mobile Network Traffic Variations

We used the Maximal Information Coefficient (MIC) [39] to identify and characterize novel associations between the adverse weather conditions and effective variations in mobile network traffic intensity. The MIC, which belongs to a larger class of Maximal Information-based Nonparametric Exploration MINE statistics [40], measures the dependence, *i.e.*, the strength, of any type of two-variable relationships. It has been tested for datasets containing, for example, socio-economic indicators or gene expression profiles. The MIC satisfies two heuristic properties: (1) generality, *i.e.*, the associations to be found are not limited to linear or non-linear functional relationships; (2) equitability, *i.e.*, ‘the statistic should give similar scores to equally noisy relationships of different types’ ([39], p. 1518). We considered both properties to be particularly important when analyzing the complex human-environmental relationship. As the sample size grows, the MIC tends to 1 ‘to all never-constant noiseless functional relationships’ ([39], p. 1520), and it converges to zero if—if and only if—the two variables are statistically independent.

For the data sets used herein the first and fixed variable was the adverse weather condition, which was valid for the entire test area. The second and altering variable was the effective variation in mobile network traffic intensity, which was computed per ‘pixel’ within the test area (see Subsection 5.4.).

## 4. Case Study Setting

Based on our previous case study [7], additional data was acquired, firstly, to validate past results, and, secondly, to focus on the urban area. The urban area is of particular interest due to its higher dynamic of human behavior compared to rural regions. This higher dynamic should be reflected in user-generated mobile network traffic.

### 4.1. Study Area and Data Sets Used

The study area comprises the urban area of the city of Udine (Figure 1). Udine, with around 100,000 inhabitants, is the old capital of the province of Friuli, located in North-Eastern Italy about 45 kilometers from the Adriatic coast.

We used environmental and mobile telecom traffic data sets for the period between 10 September and 30 September 2009 for the study area. Within the environmental data we used five meteorological parameters, namely rainfall measured in millimeter [mm], air temperature measured in degree Celsius [°C], relative humidity measured in percent [%], air pressure measured in hectopascal [hPa] (1 hPa = 1 mbar), and solar radiation measured in kilo Joule per square meter [kJ/m^2^]. These measurements were hourly averages measured by a fixed and accurately calibrated weather station, which is—in combination with others—used for weather forecasting by the regional environmental agency.

The telecom traffic data was provided fully anonymized and aggregated by Telecom Italia Mobile in raster and vector formats at 15-minute time intervals. The overall network traffic intensity is measured in Erlang and was represented as 250 m × 250 m raster data sets with full coverage of the study area. The traffic intensity value at each raster cell is the result of a model applied by the telecom operator to estimate the best serving cell to establish a mobile connection.

### 4.2. Data Consolidation and Preparation

Both datasets were consolidated on a GIS platform and linked to the same underlying space-time basis. Within a five kilometer buffer zone of the environmental monitoring station, the meteorological parameters used were assumed to be homogenous—especially for a flat region like urban Udine. These parameters were therefore considered ‘global’, *i.e.*, valid for every ‘pixel’ in the test area. For the purpose of bivariate spectral analysis, the zonal statistics of mobile phone traffic intensity within the 5 km buffer zone was calculated and then aggregated on an hourly basis. This aggregation was necessary in order to equalize the temporal resolution of meteorological data and telecom data, which is a requirement for bivariate spectral analysis. For the spatial analysis of variations within mobile phone traffic, the original spatial resolution of raw data was used (250 m × 250 m). For the temporal analysis, however, the data values of the 15-minute intervals were summed up to one hour intervals to match the time-series length of the weather data for the MIC analysis.

## 5. Results

### 5.1. Preparation for Exploratory Factor Analysis of Meteorological Variables

We considered five meteorological variables: rainfall R, air temperature AT, relative humidity RH, air pressure AP, and solar radiation SR. To ensure sufficient significant correlations among these variables we applied the Bartlett's test of sphericity [41], and the Kaiser-Meyer-Olkin (KMO) test [42]. The KMO test measures the sampling adequacy and can be interpreted as an index for comparing the magnitudes of the observed correlation coefficients to the magnitudes of the partial correlation coefficients. For the Bartlett's test, the calculated χ^2^ value of 1,481.495 with ten degrees of freedom (Table 1, with AP) is considerably higher than the critical χ^2^ value at a significance level of <0.005 (25.188). Thus, the correlation matrix is not the identity matrix, and there is significant correlation among the five variables.

The overall KMO Measure of Sampling Adequacy (MSA) of 0.646 (Table 1, with AP), indicates a sufficient overall correlation among the five variables and thus confirms the Bartlett's test. However, the individual MSA—which is shown in the main diagonal of the anti-image correlation matrix and indicates how strongly each meteorological variable correlates with all the others—pinpoints that the air pressure (0.407) is below the threshold of 0.6 (Table 2, with AP). Therefore, we excluded AP from further analysis. The subsequent recalculation of the Bartlett's test, the KMO test (both in Table 1, without AP), and the anti-image correlation matrix (Table 2, without AP), consistently approved the involvement of the four remaining variables R, AT, RH, and SR for factor analysis [42].

### 5.2. Exploratory Factor Analysis of Meteorological Variables

In order to find underlying uncorrelated factors in meteorological data we selected the Principal Component Analysis (PCA) as the factor extraction method. As shown in Table 3, 90.163% of the total variance, *i.e.*, specific, common, and error variance, in four meteorological variables can be explained by two principal components. Although the structure and relationship of the original variables is lost through the projection of the data's variance onto two principal components, both the original variables as well as the remaining principal components are intended to describe the higher level phenomenon, namely weather.

The final loadings of the two extracted principal components by the four meteorological variables are shown by the rotated component matrix (Table 4). Similar to our previous case study [7], principal component 1 is heavily positively loaded by air temperature and solar radiation, and heavily negatively loaded by relative humidity. Since this corresponds to ‘normal weather’ conditions we termed the first principal component just the same. In contrast, principal component 2 is extremely positively loaded by rain; moderately positively by relative humidity, and slightly negatively by air temperature and radiation. As these loadings indicate adverse weather conditions we termed the second principal component ‘adverse weather’.

**Figure 2.**
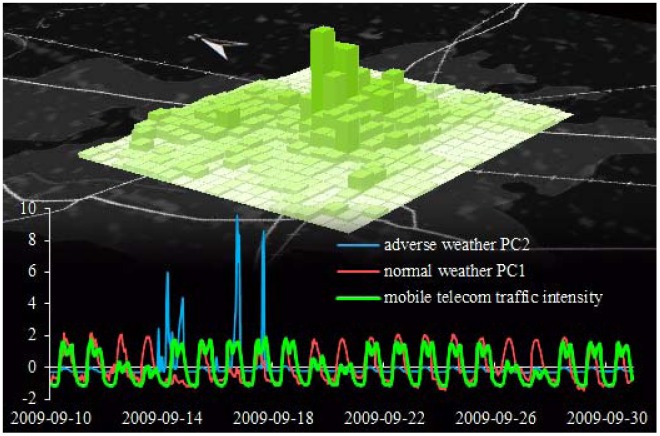
21 day-time series of total telecom traffic intensity, normal and adverse weather conditions in urban Udine; map: temporally accumulated telecom traffic intensity per 250 m ‘pixel’.

Periodic elements in the remaining time series are obvious in the time domain (Figure 2), especially for normal weather conditions (principal component 1), and mobile telecom traffic intensity. To explore such periodic patterns and their potential relationships we performed a bivariate frequency domain analysis. To ensure that the respective principal component scores used for further analyses are both standardized and uncorrelated with each other (distinction between normal and adverse weather), they were estimated using the Anderson-Rubin approach [43].

### 5.3. Spectral Analysis of Basic Weather Conditions and Mobile Telecom Traffic

In our previous work [7] we correlated basic weather conditions with mobile telecom traffic in the frequency domain to reveal periodic patterns. For the spectral analysis in this paper, we followed the same methodology but for a ten days longer time series. Emphasis was put on the sinusoidal decomposition of the ‘mobile telecom traffic intensity’ time series (Figure 2, green graph) that falls within a daily circle. The periodogram shows the main peak at a frequency of f1 = 0.0417/h (T1 = 1/f1 = 24 h), which is the first harmonic and it intuitively indicates the dominance of the day-night pattern. The second (f2 = 0.0833/h; T2 = 12 h) and third harmonics (f3 = 0.125/h; T3 = 8 h) contribute ∼6% and ∼12% of the first harmonic's magnitude, which is fully conform to our previous results in [7]. The second and third harmonics influence (depending on their phasing) the double-peak at noon in the time domain. This signifies working/non-working hours as described in more detail in [19,44].

**Figure 3.**
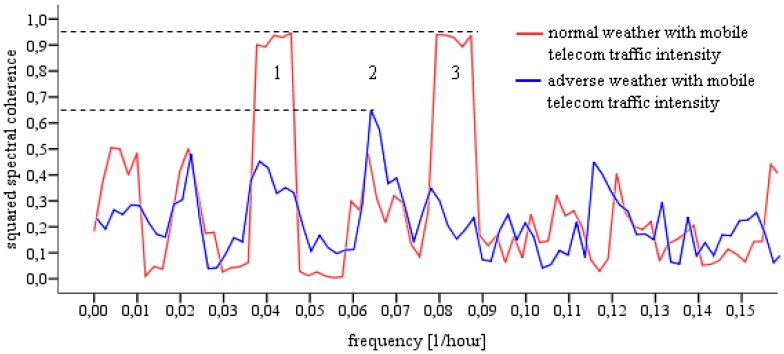
Spectral correlation of normal and adverse weather conditions with telecom traffic intensity.

For bivariate spectral analysis we consider ‘mobile telecom traffic intensity’ the dependent and ‘normal/adverse weather’ the independent variable and focus on the squared spectral coherence γ^2^—the squared magnitude of the cross-spectrum. ‘Within each frequency band, the squared coherence (like an R^2^ in regression analysis) estimates the percentage of the variance in time series X that is predictable from time series Y, within this particular frequency band’ ([38], p. 138). The result shown in Figure 3 illustrates the squared coherence of mobile network traffic intensity with normal/adverse weather conditions—this corresponds to the time series X and Y respectively cited above. Our emphasis is put on dominant periodic components in the interval 8 h ≥ T ≥ 24 h marked as 1, 2, and 3 in Figure 3. For these three components the zero coherence hypothesis, according to [45], was rejected.

### 5.4. Spatial and Temporal Extraction of Effective Variations in Mobile Network Traffic

The effective variations in user-generated mobile network traffic, which is used as a proxy for the unusual collective human behavior, were computed per spatially explicit ‘pixel’ for the entire period of 21 days in four consecutive steps:

1. Calculating the mean temporal signatures of the mobile network traffic: the mean value *x̄_h_* for each hour *h* ∈ [0, 23] was calculated (1) separately for weekends (Saturday and Sunday) and weekdays (Monday to Friday). For instance, all values at 10 AM on a weekday (or on a weekend) were averaged (the 15 minute interval values were summed up to the corresponding hour to equalize the one hour interval of the adverse weather time series as this is a precondition of the MIC computation):
  (1) $${\bar{x}}_{h}=\frac{1}{n}\sum_{i=1}^{n}x_{i}$$
  This result in two mean signatures for each ‘pixel’, one for the weekend and another one for the weekdays, each contains 24 mean values of the respective hours.
2. Calculating the differences between the mean and the actual value by distinguishing weekdays from weekends:
  (2) $$\Delta x_{i}=x_{i}-{\bar{x}}_{h}$$
3. Calculating the mean temporal signatures of the differences: this step was implemented to take into account the dominant day/night pattern within mobile network traffic, which also biased the relative variability in the differences calculated in step 2 (relatively low variability during the day, and relatively high variability during the night):
  (3) $${\bar{\Delta x}}_{h}=\frac{1}{n}\sum_{i=1}^{n}\Delta x_{i}$$
4. Calculating the effective variations *v_i_* by subtracting the mean hourly variation from the difference:
  (4) $$\upsilon_{i}=\Delta x_{i}-{\bar{\Delta x}}_{h}$$

The overall result of this four-step computation was a three-dimensional cube (2D space plus time) of effective variations, *i.e.*, minimizing the dominant day-night pattern, within user-generated mobile network traffic per ‘pixel’. These variations were then used to explore the potential relationships with adverse weather conditions.

### 5.5. Exploring Spatio-Temporal Relationships between Distinct Adverse Weather Conditions and Variations in User-Generated Mobile Network Traffic

**Figure 4.**
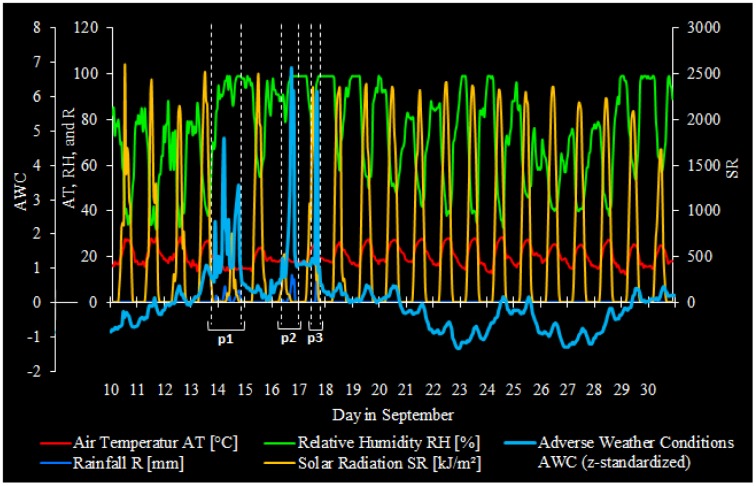
21 days of adverse weather conditions and its loading meteorological components including three distinct adverse weather periods p1, p2, and p3.

From the 21 day time series of adverse weather conditions (the second principal component) and its loading meteorological components shown in Figure 4, three distinct periods were identified: the first period (p1) is considered to be from Sunday 13 September 7 PM until Monday 14 September 10 PM (28 h in total); the second period (p2) is considered to be from Wednesday 16 September 7 AM to Thursday 17 September 0 AM (18 h in total); and the third period (p3) is considered to be from Thursday 17 September 10 AM to 9 PM (12 h in total). The adverse weather conditions were assumed to be ‘global’, *i.e.*, valid for the entire test area (as explained in Section 4.2).

For each period p1, p2, and p3, we then computed the MIC between the adverse weather conditions and the mobile telecom traffic variations for each spatially explicit ‘pixel’. The entire test area was divided into 20 longitude and 22 latitude ‘pixels’, thus resulting in a total of 440 ‘pixels’. The inherent spatial relationships between the pixels' temporal signatures was thus broken up for the MIC analysis, but remembered in individual spatial identifiers. In other words, each of the 440 ‘pixels’ was considered, on purpose, as a spatially and temporally independent entity in order to maximize the potential of disclosing a relationship between the ‘global’ adverse weather conditions and the ‘very local’ (*i.e.*, at a ‘pixel’ of 250 × 250 m) variations of user-generated mobile network traffic. This results in 440 temporal signatures of effective variations in mobile network traffic plus one signature of adverse weather conditions, thus in 440 two-variable relationships for the MIC analysis.

**Figure 5.**
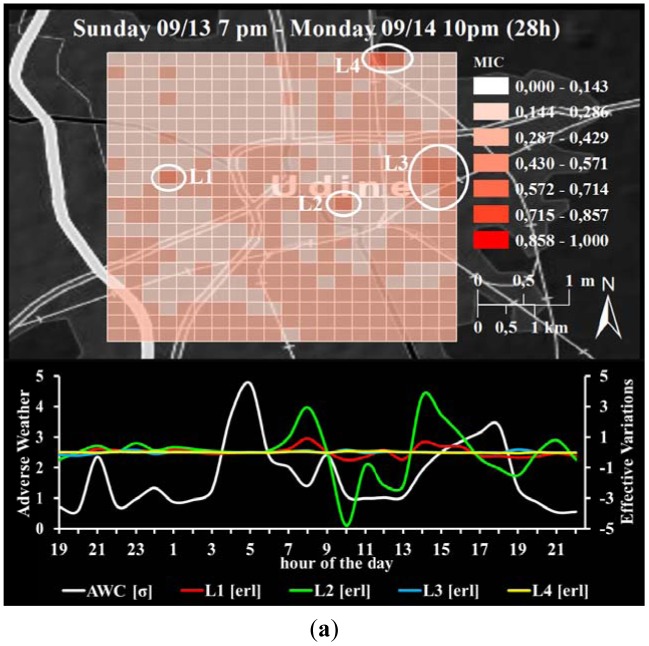

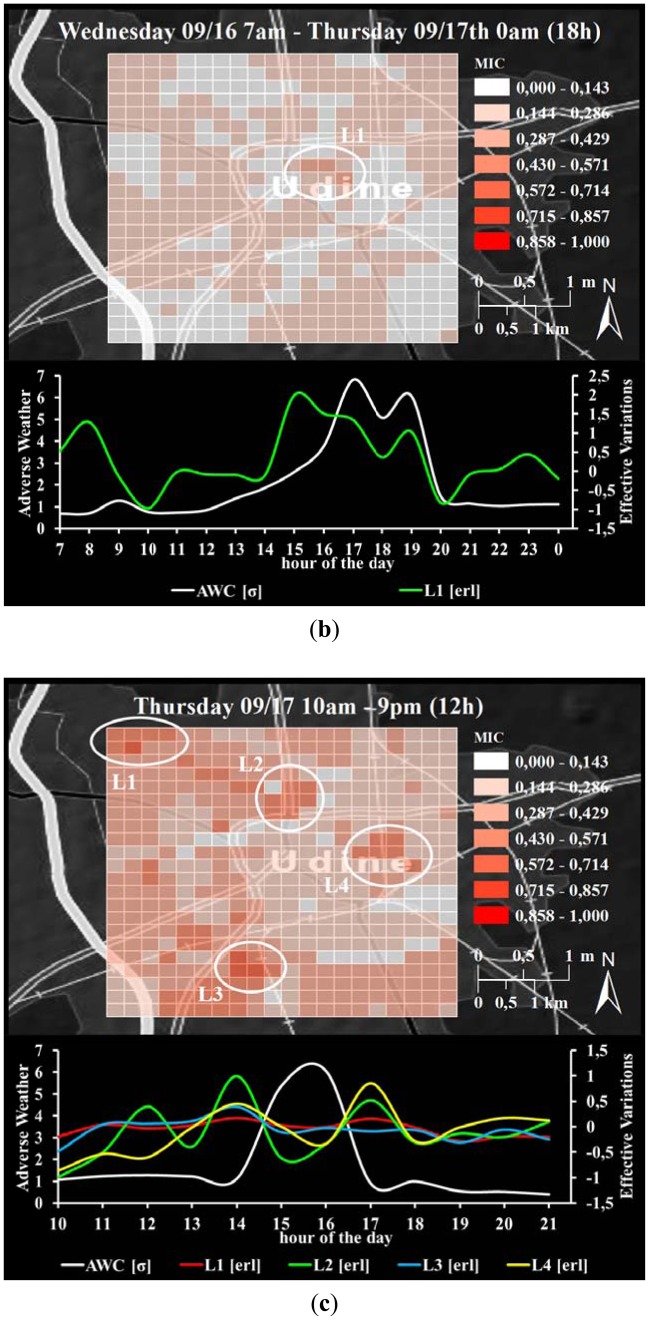
Adverse weather conditions (AWC) and effective variations in mobile network traffic: map of MICs (top), and temporal signatures of selected locations L (bottom) for the first (**a**); the second (**b**); and the third period (**c**); the temporal signatures are averaged if more than one ‘pixel’ is involved.

After the MIC computation procedure, however, the spatial relationship of the individual 440 ‘pixels’ was reconstructed into the geographic space using the aforementioned spatial identifier. This results in three maps, one per time period as shown in Figure 5(a–c). This figure shows the spatial distribution of the strength (MIC value) of the relationships between adverse weather conditions and the local effective mobile network traffic variations, as well as the two temporal signatures (note: for the bottom graphic in Figure 5(a–c), the signatures of the 440 individual variations are spatially averaged into one for the purpose of visualization only).

## 6. Discussion

The results from the explanatory factor analysis indicated that the number of meteorological variables initially used needed to be reduced from five to four, because the individual measure of sampling adequacy (MSA) of the variable air pressure against the other variables was below the threshold of 0.6. Nevertheless, this dimension reduction method can be used to extract basic weather conditions from a number of meteorological variables. The resulting two principal components represent 90.163% of the original variance while reducing 50% of the data volume. Again, we stress that both the original variables and the principal components were used to describe the higher-level phenomenon, which is the weather.

Spectral analysis of user-generated mobile network traffic clearly indicated that most of the power of the relationship between weather and mobile telecom traffic was, as previously shown in [7], centered on their harmonics. This reflects the predominant day/night pattern present in all variables considered in general (T = 24 h). In mobile telecom traffic the additional periodic patterns at T = 12 h, and 8h signify working/non-working hours within the daily circle, which agrees with [19].

Results from the bivariate spectral analysis show that normal weather (Figure 3, peak 1 and 3: γ^2^ ≤ 94%), rather than adverse weather conditions (Figure 3, peak 2: γ^2^ ≤ 63%), spectrally correlates with mobile telecom traffic at harmonics. This is in agreement with our previous findings [7] and does not reveal much new information. In contrast to our previous results, however, the relatively strong spectral correlation of mobile telecom traffic with adverse weather conditions almost disappear herein. In fact, the relatively high level of noise in the frequency domain indicates that adverse weather conditions have very low periodicity at the given temporal scale in the time-domain, which is easily recognizable in Figure 2. In order to evaluate such irregular weather conditions, or even high intensity weather extremes, and their potential relationships with variations in mobile network traffic, we put our emphasis on three clearly recognizable adverse weather periods (p1, p2, and p3 in Figure 4).

For each of these three periods we assessed the relationship between the ‘global’ adverse weather conditions and the ‘very local’ effective variations in mobile network traffic and using the maximal information coefficient MIC. Due to the inherent spatial reference of every single pixel this analysis resulted in three maps of MICs showing the strength of the aforementioned relationship (Figure 5(a–c)). Although these maps seem to represent some degree of ‘noise’ and ‘randomness’, certain patterns can be identified. We used numerous online information sources including Google Maps' Street View, Open Street Map, Wikipedia, and archives of news-sites to interpret these patterns as discussed below. At this point we want to stress that the focus of this paper is the methodology. The interpretation of these patterns is, so far, based on logical and exploratory reasoning. A thorough accuracy assessment of the results obtained is beyond the scope of this paper, it is subject of the ongoing research.

In the first and longest period p1 (28 h from Sunday evening to Monday late night), Figure 5(a) shows relatively strong relationships in the city center compared to the periphery (MICs ≈ 0.35 and ≈0.25). This could indicate that the adverse weather conditions in general and, time-delayed, at the Monday morning rush-hour time in particular (Figure 5(a) bottom, between 7 and 9 am) cause high variation in mobile phone usage. Furthermore, two locations were found with a relatively high MIC value of 0.5 (Figure 5(a) L1 and L2). The area at L1 covers a bus hub with a large parking lot—it is the ‘switchboard’ of the large public transport company SAF Autoservizi, where its urban service office is also located [46]. The area shown at L2 covers the ‘Centro Studi Volta’, a school for multidisciplinary education [47]. Both the former and the latter location intuitively imply a high degree of human dynamics, especially at the transition from the main vacation period to the beginning of a new school or academic year. Following this line of reasoning, the rather strong relationship of variations in mobile network traffic with adverse weather conditions is plausible. In the north-east part of the study area, which is dominated by residential areas, the overall strength of the relationships of ∼0.34 may indicate that people living there change their calling behavior due to the adverse weather. This might particularly apply to L3 in Figure 5(a). The highest MIC value of 0.69 (Figure 5(a) L4) in the given period was observed in an area which is a well-known and official and ‘camping’ place for nomadic people and gypsys living in poor conditions (e.g., [48]). This may indicate that the rainfall of up to 7 mm per hour triggered some preventive actions by these people to keep their home environment clean.

In the second period p2 (Figure 5(b)) the MICs show a weak overall relationships (average for the entire test area =0.17). This could indicate considerable independence of variations in network traffic from adverse weather during business hours. The small cluster at L1 with MICs of ∼0.36 in Figure 5(b) covers an area that contains the University Residence and the Cultural Center of Grace [49]. Since this location implies active human interaction, some variations in this interaction might be associated to the adverse weather conditions.

The third period p3, which covers almost only business hours, shows relatively weak relationships in the city center (MIC ≈ 0.2) as compared to the periphery (Figure 5(c)). This is in contrast to p1 (partly Sunday) but in agreement to p2 (weekdays), which could be due to the strength of the ongoing business and its considerable independence from weather conditions—people need to work in all weather. Furthermore, we identified four areas (L1 to L4 in Figure 5(c)): L1 with 0.46 ≤ MIC ≤ 0.65 is mainly dedicated to university education, whereas the outdoor area with a MIC of 0.65 is commonly used by students to meet, learn, study, socialize *etc.* According to [50], however, the new academic year had not had officially started. A comprehensible explanation of that relationship thus remains pending; L2 is dominated by a traffic junction of the busy main road entering the city from the north and the ring road. The fair MIC of 0.46 could thus be associated with problems in road traffic flow due to bad weather conditions; L3 and L4 with 0.46 ≤ MIC ≤ 0.65 is mostly residential; at L4, a double-track railway line crosses the area from north to south.

## 7. Limitations and Constraints

This approach has several limitations. Firstly, the relationship between the weather and collective human activity is highly complex and multifaceted. Several of the relationship's spatio-temporal aspects are yet unknown. For instance, where and when certain weather conditions such as heavy rainfall or extraordinary hot days influence people's activity and mobility. Thus, the data sample and analysis methods used herein are an attempt to reveal and assess some of these aspects, which are, at least, carefully examined approximations.

Secondly, the collective human activity considered was derived from user-generated traffic in the mobile network of only one operator, namely Telecom Italia Mobile (TIM). TIM has a market share of 34.2% at a mobile penetration rate of about 155% (both proportions are for the year 2010 [51]). Based on these facts we suppose that more than one third of the population's activity is reflected in the user-generated mobile network traffic. The availability of additional mobile network data would thus rather stabilize than bias the results.

Thirdly, the characteristics and patterns of human behavior are influenced by a variety of factors, ranging from socio-economic boundary conditions (week and weekends, holidays, business or school times, transportation timetables, opening times for business and government offices), social attitudes (propensity to use mobile devices for different types of activities, social segmentation of mobile users) or behavioral choices. The weather, especially adverse weather conditions, is potentially one of these influencing factors, but yet in an unknown fraction.

Another constraint was the availability of valid mobile network traffic data: The 21-day time series used in this case study is the longest continuous time period in our entire data base, where both meteorological and specifically telecom data are valid, *i.e.*, no breaks or erroneous data. This was verified by an extensive exploratory data analysis on the entire raw data set in advance. Due to the rather short time series, two issues concerning the size of the data sample need to be addressed:

- *temporal patterns in telecom traffic:* the general temporal patterns found within the telecom traffic data set used herein are conform with the patterns found in a seven-week period in Sevtsuk and Ratti [19]. The tree-week period of telecom data used herein should therefore be representative for the collective human behavior nonetheless. The length of the time series, however, affects the validity of the average signatures calculated. To reduce that effect, we aggregated the mobile traffic data from the original 15 minute time intervals to 1 h time intervals, thus stabilizing the respective average. The use of longer time series—for instance one year—of both mobile phone data and weather data would firstly allow the discrimination per day of the week (instead of ‘only’ weekday/weekend), and, secondly, enable analyses considering the seasons or holidays.
- *sample size for the Maximal Information Coefficient:* Although a ‘sufficient sample size’ is assumed in [39], a minimum sample size to achieve stable MICs is still required. Herein, the sample size of each of the variables corresponding to the three distinct adverse weather periods (refer to sub Section 5.5.) was 28 for p1, 18 for p2, and 12 for p3. Although a larger sample size would probably lead to a more stable MIC, we believe, nonetheless, that it is the most appropriate statistic available today to measure the strength of the complex environmental-human relationships.

Finally, independent of whether or not the MIC indicates relatively strong or weak relationships between effective variations in user-generated mobile network traffic and adverse weather conditions, providing definite statements about underlying spatio-temporal processes or causality is beyond the scope of this paper. Nevertheless we provide some interpretations based on logical and exploratory reasoning of the results obtained, however, the development of appropriate spatio-temporal reasoning techniques and a thorough accuracy assessment remains a tricky scientific challenge for our ongoing research.

## 8. Conclusions

In this paper we address the research need for assessing direct measures of environmental stressors on human functions by evaluating the spatio-temporal relationships between collective human dynamics and weather in an urban environment. We began with the hypothesis that a variety of environmental factors influence human behavior. Such influencing factors include, for example, pollution and noise from industrial areas and transportation networks, but also temporary effects from large public events or construction work. Most of these effects are difficult to measure in respect to their relative effects on the population or sub-groups. For simplicity purposes, the Udine study considers only one context that might influence mobile phone usage, namely the weather. To identify and characterize the relationship between weather conditions and collective human behavior, a hybrid approach which comprises several spatio-temporal statistical analyses methods was developed.

We conclude from the results discussed above that the approach presented is a promising step towards a holistic understanding of the complex relationship between environmental and social dynamics. In addition to our previous study [7], the results show that the dependence of the relationships between the ‘global’ adverse weather conditions and the effective ‘local’ variations within user-generated mobile network traffic varies spatially and temporally. For instance, it differs between city center and periphery, among some selected locations, and between weekdays and the weekend. Places with an obviously high degree of human dynamics varying over time show a higher dependence on adverse weather conditions compared to rather ‘calm’ locations. From a human point of view, this seems obvious as it is based on the experiences made and knowledge acquired in daily life. From a ubiquitous geo-sensing point of view however, such ‘experiences’ can be evaluated through context-aware analysis. In the interpretation of the results we generated some hypotheses and provided some possible answers to them. The results, as well as their interpretation, by no means reveal causality, however, they suggest that the variations in collective human behavior in the context of the ‘global’ adverse weather must have different underlying drivers. These drivers depend on the functional configuration of the urban environment.

Further, we showed that factor analysis can be used to halve the amount of data needed for further analysis while keeping more than 90% of its original information. In the context of (near) real-time data streams from an increasing variety of ubiquitous sensing sources, this can significantly decrease processing demands and thus enhance the performance of near real-time analyses. Being aware of the limitation and constraints of this approach, we nevertheless demonstrated that novel and cross-domain analysis methods and statistics such as the MIC can be used to explore, from a ubiquitous sensing perspective, the human-environment interface, e.g., the effect of adverse weather on collective human behavior. Referring back to the research question stated at the beginning, our approach provides some novel cross-domain analysis methods to examine collective human behavioral patterns considering the environmental context, in this case basic weather conditions.

This research aims to contribute to a better understanding of the complex human-environmental relationship in three ways: Firstly, we demonstrated that the consolidation of data from ubiquitous sensing technologies on a common space-time basis enabled a context-aware analysis of environmental and social dynamics. Herein we used the example of environmental sensor networks and mobile communication networks as ‘large-scale’ sensors. Secondly, we showed that the application of interdisciplinary analysis methods from the space-, time-, and frequency domain can identify and characterize known and novel relationships between environmental and social dynamics. Thirdly, as a potential far-reaching impact, we believe that the overall approach presented can thus allow for a holistic understanding of physical and social dynamics and their underlying spatio-temporal processes.

Our ongoing research activities focus on the verification of the results obtained herein. The thorough accuracy assessment will therefore integrate additional data sources. For instance, traffic data of individual and public transport, records of road construction and maintenance, event records of emergency services (police, fire, and ambulance), census data, detailed land use data, mobile network traffic of another operator, historical social media data (e.g., Facebook and Twitter), as well as the statements from people who are familiar with the dynamics of the city (e.g., traffic researchers or locals). From a methodological point of view, we may also consider alternative statistics to further characterize the human-environmental relationship. Therefore, as stated in [52], the distance correlation measure [53] is a promising candidate. These tasks are intended to support the identification of causality within the relationship between weather conditions and collective human behavior: e.g., why is there a strong/weak relationship?; is there any specific underlying feature or process which may have a spatial and/or temporal component? Hence, to answer such questions, new scientific challenges arise which need to be solved.

## Figures and Tables

**Table 1.** Kaiser Meyer Olkin and Bartlett's Test of meteorological variables, with and without air pressure (AP).

		**with AP**	**without AP**
Kaiser-Meyer-Olkin Measure of Sampling Adequacy		0.646	**0.703**
Bartlett's Test of Sphericity	Approx. Chi-Square	1,481.495	**1,266.950**
	degrees of freedom	10	**6**
	significance	0.000	**0.000**

**Table 2.** Anti-image correlation matrix: including air pressure (left), without air pressure (right).

	**R**	**AT**	**RH**	**AP**	**SR**		**R**	**AT**	**RH**	**SR**
R	**0.770** ^a^	0.009	−0.051	0.232	−0.010	R	**0.646** ^a^	−0.079	−0.205	−0.01
AT	0.009	**0.607** ^a^	0.733	0.349	−0.535	AT	−0.079	**0.640** ^a^	0.689	−0.571
RH	−0.051	0.733	**0.645** ^a^	0.517	0.025	RH	−0.205	0.689	**0.716** ^a^	0.03
AP	0.232	0.349	0.517	**0.407** ^a^	0.000	SR	−0.01	−0.571	0.03	**0.785 ^a^**.
SR	−0.010	−0.535	0.025	0.000	**0.808** ^a^					

a Measures of Sampling Adequacy (MSA).

**Table 3.** Total variance of two principal components extracted from four meteorological variables.

**Princ. Comp.**	**Initial Eigenvalues**			**Extraction Sums of Squared Loadings**			**Rotation Sums of Squared Loadings**		

	**Tot.**	**Var. %**	**Cumulative %**	**Tot.**	**Var. %**	**Cumulative %**	**Tot.**	**Var. %**	**Cumulative %**
1	2.647	66.173	66.173	2.647	66.173	66.173	2.583	64.569	**64.569**
2	0.960	23.990	90.163	0.960	23.990	90.163	1.024	25.594	**90.163**
3	0.282	7.060	97.223						
4	0.111	2.777	100.000						

**Table 4.** Loadings of the four meteorological variables on the two principal components.

**Original Variable**	**PC 1**	**PC 2**
rainfall R	−0.086	0.995
air temperature AT	0.960	−0.064
relative humidity RH	−0.909	0.173
solar radiation SR	0.909	−0.019

Rotation Method: Varimax with Kaiser Normalization.
